# Hemodynamic and Vascular Stressor Exposure and Outcomes Among Inpatient Hospitalization with Chronic Kidney Disease: A Nationwide Study

**DOI:** 10.3390/jcm15124747

**Published:** 2026-06-18

**Authors:** Brent Tai, Chijioke Okonkwo, Yaroslav Zuyev, Derek Snyder

**Affiliations:** Department of Internal Medicine, BayCare Health System, Clearwater, FL 33759, USA; chijioke.okonkwo@baycare.org (C.O.); yaroslav.zuyev@baycare.org (Y.Z.); derek.snyder@baycare.org (D.S.)

**Keywords:** chronic kidney disease, hospitalization, in-hospital mortality, acute kidney injury, sepsis, heart failure, hemodynamic stress, vascular disease, risk stratification, National Inpatient Sample

## Abstract

**Background:** Hospitalized adults with chronic kidney disease (CKD) experience high morbidity and mortality. Acute inpatient events frequently occur in combination, yet most studies evaluate individual conditions in isolation. Acute hemodynamic and vascular stressors may represent interacting physiological stressors that define heterogeneous patterns of inpatient risk. **Methods:** Acute hemodynamic stressors (sepsis, shock, acute decompensated heart failure, and mechanical ventilation) and vascular stressors (acute myocardial infarction, major bleeding, stroke, pulmonary embolism, and deep vein thrombosis) were identified using ICD-10-CM and ICD-10-PCS codes. Stressor burden was defined as the number of stressors (0, 1, 2, or ≥3). Hospitalizations were categorized into mutually exclusive domains: none, hemodynamic only, vascular only, or both. Survey-weighted multivariable regression models examined associations with mortality, acute kidney injury (AKI), length of stay (LOS), and hospital charges. Prespecified sensitivity analyses excluded inter-hospital transfers, and interaction analyses assessed modification by age. **Results:** Among 1,062,813 CKD hospitalizations, 66.1% experienced at least one acute stressor. Increasing stressor burden demonstrated a marked dose–response relationship with mortality, with adjusted odds ratios of 2.15 (95% CI: 2.08–2.23), 7.36 (95% CI: 7.09–7.64), and 31.65 (95% CI: 30.40–32.95) for 1, 2, and ≥3 stressors, respectively. Increasing stressor burden was also associated with higher odds of AKI, longer LOS, and greater hospital charges. Significant dose–response relationships were observed for all outcomes (all P-trend < 0.001). Isolated hemodynamic stressors were associated with greater mortality risk than isolated vascular stressors (aOR: 4.97 vs. 2.15), while hospitalizations experiencing both domains had the greatest risk (aOR: 13.10, 95% CI: 12.52–13.71). These findings were robust in sensitivity analyses excluding inter-hospital transfers. The relative increase in mortality associated with higher stressor burden was greater among patients younger than 65 years than among older adults (P for interaction <0.001). **Conclusions:** Acute hemodynamic and vascular stressors define heterogeneous patterns of inpatient risk among hospitalized adults with CKD. Both cumulative stressor burden and stressor domain are strongly associated with mortality, AKI, and resource utilization, with robust dose–response relationships that highlight acute physiological stress as an important determinant of inpatient outcomes in CKD.

## 1. Introduction

Chronic kidney disease (CKD) is highly prevalent among hospitalized adults and is associated with substantial inpatient morbidity, mortality, and healthcare utilization [1,2,3]. Patients with CKD experience frequent hospitalizations and are particularly vulnerable to acute decompensation due to impaired physiological reserves, high comorbidity burden, and altered cardiovascular and hemostatic function [4,5,6]. As a result, hospitalizations involving CKD account for a disproportionate share of adverse inpatient outcomes and resource use, underscoring the need for improved frameworks to understand and stratify inpatient risk in this population.

Prior studies of inpatient outcomes in CKD have largely focused on individual acute conditions, such as sepsis, acute kidney injury, heart failure exacerbations, or acute myocardial infarction [7,8,9,10]. While these studies have provided important insights, they typically examine acute events in isolation and evaluate outcomes one at a time. Such single-condition frameworks may inadequately capture the complexity of inpatient illness, particularly among patients with CKD, in whom multiple acute processes frequently unfold concurrently during hospitalization.

In clinical practice, acute inpatient events rarely occur in isolation. Hospitalized patients with CKD often experience multiple simultaneous physiological insults, including circulatory instability, cardiac decompensation, infection, bleeding, and thromboembolic or cerebrovascular events [11,12,13]. These co-occurring stressors may interact synergistically, leading to abrupt deterioration that is not fully explained by any single diagnosis. Despite this reality, there remains a lack of integrative approaches that explicitly account for the cumulative and interacting nature of acute inpatient stressors in CKD.

Conceptually, acute inpatient stressors can be broadly categorized into hemodynamic and vascular domains. Hemodynamic stressors reflect systemic circulatory or cardiopulmonary instability, including sepsis, shock, acute decompensated heart failure, and mechanical ventilation. Vascular stressors include acute myocardial infarction, major bleeding, stroke, pulmonary embolism, and deep vein thrombosis. These domains differ in underlying pathophysiology and may confer distinct patterns of risk and resource utilization, particularly in patients with CKD, who are uniquely susceptible to both circulatory instability and vascular events.

Beyond the presence or absence of individual stressors, both the cumulative burden and the qualitative domain of stressors may be clinically meaningful. Increasing numbers of concurrent stressors may signal escalating physiological instability, while the combination of hemodynamic and vascular stressors may represent a distinct and particularly high-risk inpatient phenotype. Moving beyond binary event-based frameworks toward approaches that capture both stressor burden and stressor domain may therefore provide a more nuanced understanding of inpatient risk in CKD. Rather than evaluating individual acute diagnoses in isolation, this study introduces an integrative stressor-based framework that captures both the cumulative burden and qualitative domain of acute inpatient physiological stress among hospitalized adults with CKD. This approach reflects how acute illness unfolds in clinical practice and provides a clinically interpretable lens for inpatient risk stratification.

Accordingly, we conducted a nationwide analysis of hospitalized adults with CKD to (1) characterize the prevalence and co-occurrence of acute hemodynamic and vascular stressors; (2) examine their associations with in-hospital mortality, acute kidney injury, length of stay, and hospital charges; (3) evaluate dose–response relationships according to cumulative stressor burden and qualitative stressor domain; and (4) assess the robustness of these associations using prespecified sensitivity and interaction analyses.

## 2. Methods

### 2.1. Study Design and Data Source

We conducted a nationwide, retrospective observational study using data from the 2022 National Inpatient Sample (NIS), part of the Healthcare Cost and Utilization Project sponsored by the Agency for Healthcare Research and Quality. The NIS is a stratified, discharge-level database that represents approximately 20% of all U.S. hospitalizations and enables the generation of national estimates through the application of discharge weights [14]. The database includes patient demographics, diagnoses, procedures, hospital characteristics, and outcomes. Because the NIS contains de-identified data, this study was exempted from institutional review board review by the BayCare Health System.

### 2.2. Study Population and Exposure Definitions

We included adult hospitalizations (aged ≥18 years) with a diagnosis of chronic kidney disease (CKD), identified using ICD-10-CM diagnosis codes in any diagnosis position. The unit of analysis was the hospitalization rather than the individual patient.

The primary exposures of interest were acute hemodynamic and vascular stressors occurring during hospitalization. Hemodynamic stressors included sepsis, shock, acute decompensated heart failure, and mechanical ventilation. Vascular stressors included acute myocardial infarction, major bleeding, stroke, pulmonary embolism, and deep vein thrombosis. Stressors were identified using ICD-10-CM diagnosis and procedure codes and were conceptualized as markers of acute inpatient physiological stress rather than etiological causes. Stressors were treated as co-occurring inpatient events and were not assumed to follow a specific temporal sequence.

For each hospitalization, cumulative stressor burden was defined as the total number of acute hemodynamic and vascular stressors present and categorized as 0, 1, 2, or ≥3 stressors. Hospitalizations were also classified into mutually exclusive stressor domains: no stressors, hemodynamic stressors only, vascular stressors only, or combined hemodynamic and vascular stressors. The complete ICD-10-CM and procedure codes used in this study are listed in Supplementary Table S1.

Table 1 shows the distribution of individual hemodynamic and vascular stressors, stressor domains, and cumulative stressor burden among hospitalized adults with chronic kidney disease. Values are presented as unweighted counts (n) and survey-weighted percentages with 95% confidence intervals.

### 2.3. Outcomes and Covariates

The primary outcome was in-hospital mortality. Secondary outcomes included acute kidney injury (AKI), length of stay (LOS), and total hospital charges. AKI was identified using ICD-10-CM diagnosis codes recorded during hospitalization. Because AKI was identified using administrative codes, milder or transient episodes may be under-ascertained, and identified cases likely reflect clinically significant AKI. LOS and total charges were obtained from NIS variables and analyzed as continuous outcomes, with log transformation applied to address right-skewed distributions.

Covariates were selected a priori based on clinical relevance and prior literature and included patient demographics (age, sex, race/ethnicity, median household income quartile by ZIP code, and primary expected payer), hospital characteristics (region, teaching status, bed size, and ownership), and admission characteristics (elective admission and inter-hospital transfer status).

### 2.4. Statistical Analysis

All analyses accounted for the complex survey design of the NIS, including stratification, clustering, and discharge weights, to generate nationally representative estimates. Descriptive analyses compared baseline characteristics across stressor burden categories using survey-weighted means and percentages with 95% confidence intervals.

Multivariable survey-weighted regression models were used to examine associations between stressor burden and stressor domain with inpatient outcomes. Logistic regression models were used to model in-hospital mortality and AKI, and linear regression models with log-transformed outcomes were used to model LOS and total charges. All multivariable models were adjusted for age, sex, race/ethnicity, primary payer, ZIP code income quartile, hospital region, teaching status, hospital bed size, hospital ownership, elective admission status, and inter-hospital transfer status. Results are reported as adjusted odds ratios or ratios with 95% confidence intervals. Standard survey adjustment methods were applied to handle strata containing a single primary sampling unit.

To evaluate dose–response relationships, stressor burden was additionally modeled as an ordinal variable (0, 1, 2, and ≥3 stressors), and tests for linear trend were performed for mortality, acute kidney injury, length of stay, and hospital charges.

Prespecified sensitivity analyses repeated the primary models after excluding inter-hospital transfers. A prespecified interaction analysis evaluated effect modification by age group (<65 vs. ≥65 years) for the association between stressor burden and in-hospital mortality. All analyses were performed using R statistical software (R Foundation for Statistical Computing, Vienna, Austria, version 4.5.3). Two-sided *p* values <0.05 were considered statistically significant.

## 3. Results

### 3.1. Study Cohort and Distribution of Acute Stressors

A total of 1,062,813 hospitalizations involving adults with chronic kidney disease (CKD) were included, representing approximately 26.5 million hospitalizations nationally after application of survey weights. Acute hemodynamic and/or vascular stressors were common, occurring in 66.1% of hospitalizations, while 33.9% had no identifiable acute stressors (Table 1).

Hemodynamic stressors predominated. The most common individual stressor was acute decompensated heart failure (47.0%, 95% CI: 46.7–47.2), followed by sepsis (14.8%), major bleeding (10.6%), and acute myocardial infarction (8.6%). Mechanical ventilation and circulatory shock occurred in 4.6% and 3.3% of hospitalizations, respectively. In contrast, vascular stressors were less frequent, including stroke (3.4%), deep vein thrombosis (3.1%), and pulmonary embolism (1.5%).

When categorized by domain, 58.8% of hospitalizations involved isolated hemodynamic stressors, 2.8% involved isolated vascular stressors, and 4.4% involved stressors spanning both domains. In terms of cumulative burden, 43.9% had a single stressor, 16.0% had two stressors, and 6.2% had three or more stressors.

### 3.2. Baseline Characteristics and Unadjusted Outcomes by Stressor Burden

Baseline demographic, clinical, and hospital characteristics varied substantially across stressor burden categories (Table 2). The mean age increased from 68.8 years (95% CI: 68.6–69.0) among hospitalizations without stressors to 72.3 years (95% CI: 72.1–72.4) among those with two stressors, with a slight decline among those with three or more stressors (70.8 years; 95% CI: 70.6–71.0). The proportion of female patients decreased from 47.9% among hospitalizations without stressors to 42.4% among those with three or more stressors.

Table 2 shows the baseline demographic, clinical, and hospital characteristics of hospitalized adults with chronic kidney disease by hemodynamic and vascular stressor burden. Values are presented as unweighted counts (n) and survey-weighted means or percentages with 95% confidence intervals. Stressor burden was categorized as 0, 1, 2, or ≥3 acute hemodynamic and/or vascular stressors.

Unadjusted outcomes worsened substantially in a stepwise fashion. In-hospital mortality increased from 1.2% among hospitalizations without stressors to 2.8% with one stressor, 9.0% with two stressors, and 28.8% with three or more stressors. Similarly, AKI increased from 40.1% to 67.8%.

### 3.3. Stressor Co-Occurrence Patterns and Outcome-Specific Signatures

**Figure 1:**
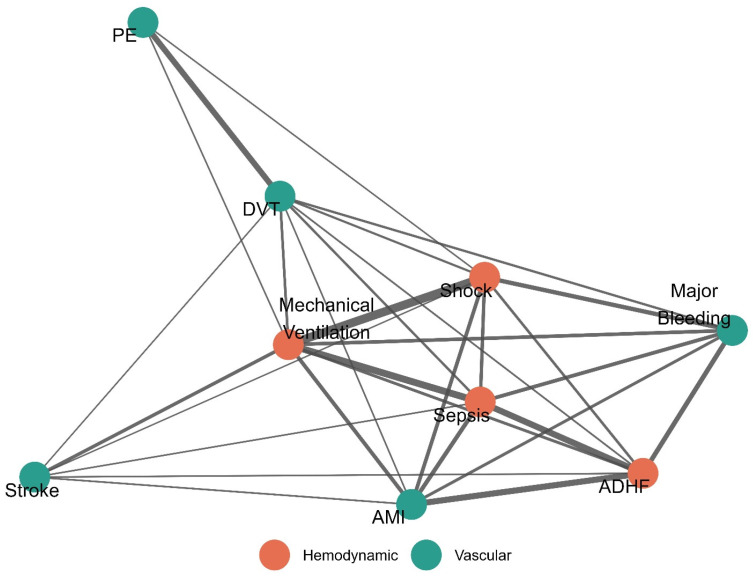
Co-occurrence network of hemodynamic and vascular stressors in hospitalized adults with chronic kidney disease.

Acute stressors frequently co-occurred rather than occurring in isolation. Network analysis demonstrated a densely interconnected core of hemodynamic stressors, with vascular stressors forming a smaller but distinct subnetwork (Figure 1).

Figure 1 is a network visualization depicting the co-occurrence of acute hemodynamic and vascular stressors among hospitalized adults with chronic kidney disease. Nodes represent individual stressors, and edges represent normalized co-occurrence strength (Jaccard similarity) across hospitalizations, with thicker edges indicating more frequent co-occurrence. Hemodynamic stressors (sepsis, shock, acute decompensated heart failure, and mechanical ventilation) formed a densely interconnected core, whereas vascular stressors (acute myocardial infarction, major bleeding, stroke, pulmonary embolism, and deep vein thrombosis) formed a distinct but connected subnetwork, highlighting heterogeneous yet interacting patterns of inpatient physiological stress.

**Figure 2:**
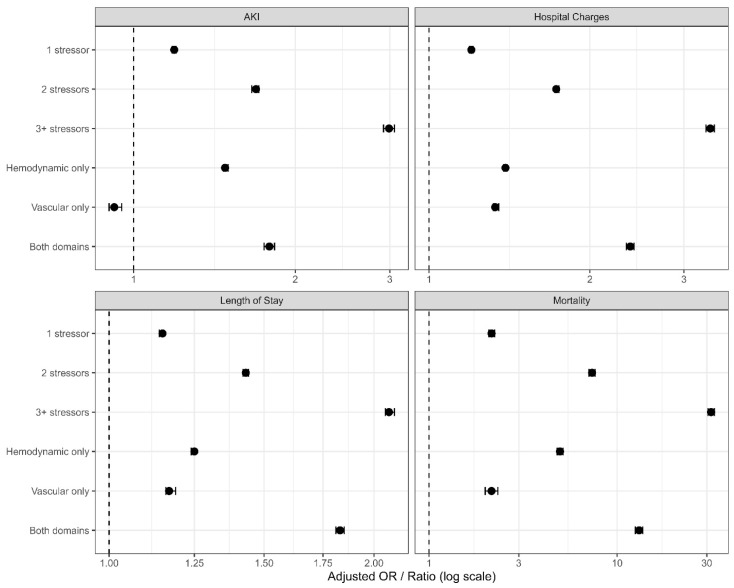
Adjusted associations of stressor burden and stressor domain with inpatient outcomes among hospitalized adults with chronic kidney disease.

Outcome associations varied substantially by stressor type (Figure 2). Hemodynamic stressors demonstrated stronger associations with in-hospital mortality and resource utilization. Vascular stressors exhibited more heterogeneous outcome profiles and a lower adjusted mortality risk than isolated hemodynamic stressors (aOR: 2.15 vs. 4.97), whereas combined hemodynamic and vascular stressors demonstrated the greatest risk across outcomes.

Figure 2 shows adjusted odds ratios (mortality and acute kidney injury) and adjusted ratios (length of stay and hospital charges) with 95% confidence intervals. Stressor burden categories (1, 2, and ≥3 stressors) and stressor domains (hemodynamic only, vascular only, and both domains) are compared with hospitalizations without stressors.

### 3.4. Adjusted Associations, Dose–Response Relationships, and Robustness Analyses

**Figure 3:**
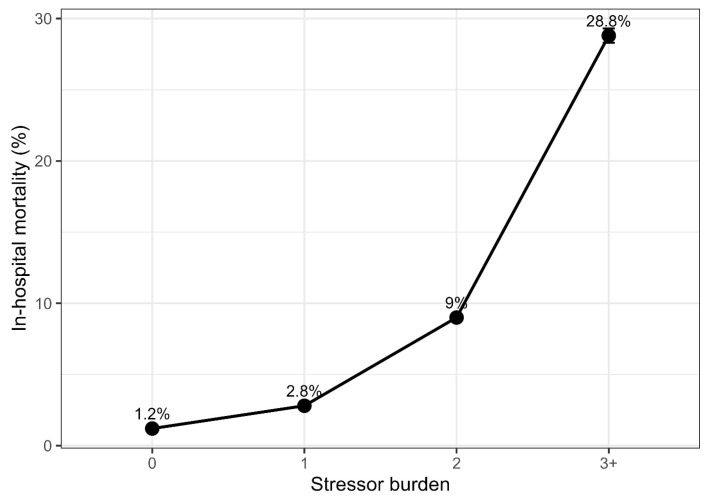
Dose–response relationship between cumulative hemodynamic and vascular stressor burden and in-hospital mortality among hospitalized adults with chronic kidney disease.

After multivariable adjustment, increasing stressor burden remained strongly associated with adverse inpatient outcomes (Table 3). Compared with hospitalizations without stressors, the adjusted odds of in-hospital mortality increased progressively with one stressor (aOR: 2.15; 95% CI: 2.08–2.23), two stressors (aOR: 7.36; 95% CI: 7.09–7.64), and three or more stressors (aOR: 31.65; 95% CI: 30.40–32.95), demonstrating a marked dose–response relationship (Figure 3).

Table 3 shows the adjusted associations between hemodynamic and vascular stressors and in-hospital outcomes among hospitalized adults with chronic kidney disease. Adjusted odds ratios (aORs) and ratios with 95% confidence intervals are shown for stressor burden and stressor domain. Models were estimated using survey-weighted regression and adjusted for demographic, clinical, and hospital characteristics.

Figure 3: Mortality rates and 95% confidence intervals are shown according to stressor burden categories (0, 1, 2, and ≥3 stressors). Mortality increased in a stepwise fashion from 1.2% among hospitalizations without stressors to 28.8% among those with three or more concurrent stressors, demonstrating a pronounced dose–response relationship.

Significant dose–response relationships were observed across all outcomes. Each one-category increase in stressor burden was associated with higher odds of in-hospital mortality (OR: 3.42, 95% CI: 3.38–3.47) and acute kidney injury (OR: 1.35, 95% CI: 1.34–1.36), as well as a longer length of stay (ratio: 1.24, 95% CI: 1.23–1.24) and greater hospital charges (ratio: 1.40, 95% CI: 1.40–1.41) (all P-trend <0.001; Supplementary Table S2).

Higher stressor burden was also associated with substantially greater resource utilization. Length of stay increased progressively with one stressor (ratio: 1.15, 95% CI: 1.14–1.15), two stressors (ratio: 1.43, 95% CI: 1.42–1.44), and three or more stressors (ratio: 2.08, 95% CI: 2.06–2.11). Similarly, hospital charges increased from a ratio of 1.20 (95% CI: 1.19–1.21) for one stressor to 3.36 (95% CI: 3.30–3.42) for three or more stressors. Increasing stressor burden was also associated with higher odds of acute kidney injury, with adjusted odds ratios of 1.19 (95% CI: 1.18–1.20), 1.69 (95% CI: 1.66–1.71), and 2.99 (95% CI: 2.92–3.06) for one, two, and three or more stressors, respectively.

Domain-based analyses revealed important qualitative differences beyond cumulative burden alone. Compared with hospitalizations without stressors, isolated hemodynamic stressors were associated with a nearly five-fold increase in mortality risk (aOR: 4.97, 95% CI: 4.80–5.16), whereas isolated vascular stressors were associated with a more modest increase (aOR: 2.15, 95% CI: 1.99–2.32). Hospitalizations experiencing both hemodynamic and vascular stressors had the greatest risk across outcomes, including mortality (aOR: 13.10, 95% CI: 12.52–13.71), acute kidney injury (aOR: 1.79, 95% CI: 1.75–1.83), length of stay (ratio: 1.83, 95% CI: 1.81–1.85), and hospital charges (ratio: 2.38, 95% CI: 2.34–2.42).

Findings were robust across prespecified sensitivity analyses. Excluding inter-hospital transfers yielded materially similar results, with adjusted odds ratios for mortality of 2.17 (95% CI: 2.08–2.26), 7.77 (95% CI: 7.44–8.11), and 34.99 (95% CI: 33.45–36.61) for hospitalizations with one, two, and three or more stressors, respectively (Supplementary Table S3). Similar patterns were observed for acute kidney injury, length of stay, and hospital charges, supporting the robustness of the primary findings.

A prespecified interaction analysis demonstrated significant effect modification by age (P for interaction of <0.001). The relative increase in mortality associated with higher stressor burden was greater among patients younger than 65 years, with adjusted odds ratios of 2.96 (95% CI: 2.69–3.26), 14.59 (95% CI: 13.26–16.06), and 67.68 (95% CI: 61.53–74.44) for one, two, and three or more stressors, respectively. Among patients aged 65 years or older, the corresponding adjusted odds ratios were 2.05 (95% CI: 1.97–2.13), 6.43 (95% CI: 6.18–6.70), and 25.59 (95% CI: 24.51–26.72), indicating a steeper relative increase in mortality risk among younger adults with increasing stressor burden (Supplementary Table S4).

## 4. Discussion

In this nationwide analysis of hospitalized adults with chronic kidney disease (CKD), we demonstrate that acute hemodynamic and vascular stressors are common and strongly associated with adverse inpatient outcomes, extending prior work that has documented the high morbidity and mortality burden of CKD hospitalizations [15]. Rather than reflecting a single severity gradient, these stressors clustered into distinct patterns, exhibited heterogeneous outcome-specific associations, and produced a nonlinear escalation in risk with increasing cumulative burden. Together, these findings highlight acute inpatient physiological stress as a central and underrecognized determinant of outcomes among patients with CKD.

A key finding of this study is the pronounced dose–response relationship between cumulative stressor burden and adverse inpatient outcomes. Increasing numbers of concurrent stressors were associated with progressively higher adjusted odds of mortality, acute kidney injury, prolonged hospitalization, and greater hospital charges. Mortality risk increased markedly across stressor burden categories, reaching more than a 30-fold increase among hospitalizations with three or more concurrent stressors. Furthermore, formal trend analyses demonstrated significant dose–response relationships for mortality, acute kidney injury, length of stay, and hospital charges. These findings support the concept that the burden of acute physiological stress is associated with a progressive escalation in risk, accelerating disproportionately once multiple acute insults co-occur [16]. Stressor burden therefore appears to function as a clinically meaningful summary measure of acute physiological instability that is not fully captured by traditional comorbidity counts or chronic disease severity measures.

From a clinical perspective, stressor burden may serve as a practical risk awareness construct rather than a predictive score. Recognition that patients with CKD experiencing multiple concurrent stressors—particularly across hemodynamic and vascular domains—are transitioning into a high-risk physiologic state may help contextualize deterioration risk and inform monitoring intensity or early multidisciplinary involvement.

Beyond cumulative burden, the qualitative nature of stressors also mattered. Isolated hemodynamic stressors were associated with substantially greater mortality risk than isolated vascular stressors, while hospitalizations experiencing both hemodynamic and vascular stressors demonstrated the highest risk across all outcomes. These findings suggest that acute circulatory and cardiopulmonary instability may represent a particularly high-risk pathway of inpatient deterioration in CKD. The markedly elevated risk observed among hospitalizations spanning both domains supports the concept that concurrent hemodynamic and vascular insults define a distinct and especially vulnerable inpatient phenotype [17,18]. These domain-based differences were visually reinforced by the co-occurrence structure of stressors and by outcome-specific association patterns, underscoring that acute inpatient events represent distinct patterns of inpatient physiological stress rather than interchangeable markers of illness. Vascular stressors, although less common, were associated with substantial resource utilization, consistent with prior studies of thromboembolic and cerebrovascular events in CKD populations, whereas hemodynamic stressors were more directly tracked with mortality risk [19].

Age-based interaction analyses revealed an important effect modification. The relative increase in mortality associated with higher stressor burden was steeper among patients younger than 65 years compared with older adults. This pattern likely reflects differences in baseline risk and physiological reserve, whereby younger patients experience a sharper relative deterioration when exposed to multiple acute insults, whereas older patients carry a higher baseline risk with a more attenuated relative escalation [20,21]. These findings emphasize the distinction between relative and absolute risk and suggest that stressor burden may have different prognostic implications across age groups.

Our findings were robust across prespecified sensitivity analyses. Excluding inter-hospital transfers yielded materially similar estimates for mortality, acute kidney injury, length of stay, and hospital charges, with effect sizes that were generally unchanged or slightly stronger than those observed in the primary analysis. These findings suggest that the observed associations were not driven by referral patterns or transfer-related bias and reinforce the stability and generalizability of the stressor burden framework.

Prior studies in CKD populations have largely focused on individual conditions such as sepsis, acute kidney injury, or heart failure in isolation [10,11,17,22]. While informative, these approaches do not fully capture the complexity of acute inpatient physiology, in which multiple stressors frequently co-occur and interact during hospitalization. By integrating co-occurrence structure, mechanistic outcome signatures, cumulative burden, and domain-based risk profiles, our study provides a comprehensive framework for understanding acute inpatient stress among patients with CKD.

The clinical and health system implications of these findings are noteworthy. Stressor burden and stressor domain may serve as early warning constructs to identify hospitalized patients with CKD at heightened risk for deterioration, prolonged hospitalization, or high resource use. Recognizing patients with multiple concurrent stressors, particularly those spanning both hemodynamic and vascular domains, may inform decisions regarding monitoring intensity, escalation of care, or early multidisciplinary involvement. While these findings are hypothesis-generating, they suggest a potential role for stressor-based frameworks in inpatient risk stratification and care planning.

Several limitations merit consideration. This study relied on administrative data, and stressors were identified using diagnosis and procedure codes, which may be subject to misclassification. The NIS lacks detailed physiologic measurements and the precise timing of events, precluding assessment of temporal relationships among stressors. It is not possible to determine whether individual stressors preceded, followed, or occurred concurrently with AKI or other outcomes. Accordingly, observed associations should be interpreted as reflecting patterns of risk rather than causal effects of individual stressors. The unit of analysis was hospitalization rather than the individual patient, and residual confounding is possible despite extensive adjustment. Nonetheless, the consistency of findings across figures and sensitivity analyses supports the validity of the observed associations.

## 5. Conclusions

In conclusion, acute hemodynamic and vascular stressors define heterogeneous and clinically meaningful patterns of inpatient risk among hospitalized adults with CKD. Both the cumulative burden and qualitative domain of stressors are strongly associated with mortality and resource utilization, with robust dose–response relationships and integrated risk profiles that persist across sensitivity analyses. These results highlight the importance of acute physiological stress in shaping inpatient outcomes and provide a foundation for future studies aimed at integrating stressor-based frameworks into clinical risk assessment and care delivery.

## Figures and Tables

**Table 1:** Prevalence and distribution of hemodynamic and vascular stressors among hospitalized adults with chronic kidney disease.

Characteristic	Unweighted n	Weighted % (95% CI)
**Individual stressors**		
Sepsis	157,667	14.8% (14.6–15.0)
Circulatory shock	35,030	3.3% (3.2–3.4)
Mechanical ventilation	48,588	4.6% (4.5–4.7)
Major bleeding	113,039	10.6% (10.5–10.8)
Acute myocardial infarction	91,878	8.6% (8.4–8.9)
Acute decompensated heart failure	498,992	47.0% (46.7–47.2)
Stroke	35,670	3.4% (3.3–3.4)
Pulmonary embolism	15,442	1.5% (1.4–1.5)
Deep vein thrombosis	32,823	3.1% (3.0–3.2)
**Stressor domain overlap**		
None	360,445	33.9 (33.7–34.2)
Hemodynamic only	625,080	58.8 (58.6–59.1)
Vascular only	30,283	2.8 (2.8–2.9)
Both	47,005	4.42% (4.4–4.5)
**Stressor burden**		
0 stressors	360,445	33.9 (33.7–34.2)
1 stressor	467,099	43.9 (43.8–44.1)
2 stressors	169,553	16.0 (15.8–16.1)
3+ stressors	65,716	6.2% (6.1–6.3)

**Table 2:** Baseline characteristics of hospitalized adults with chronic kidney disease by hemodynamic and vascular stressor burden.

Characteristic	0 Stressors	1 Stressor	2 Stressors	3+ Stressors
Unweighted hospitalizations	360,445	467,099	169,553	65,716
**Demographics**				
Age, mean (95% CI)	68.8 (68.6–69.0)	71.9 (71.7–72.0)	72.3 (72.1–72.4)	70.8 (70.6–71.0)
Female sex	47.9% (47.7–48.1)	46.6% (46.4–46.8)	44.6% (44.3–44.9)	42.4% (42.0–42.8)
In-hospital mortality	1.2% (1.2–1.3)	2.8% (2.7–2.9)	9.0% (8.8–9.2)	28.8% (28.3–29.3)
Acute kidney injury	40.1% (39.7–40.4)	45.3% (44.9–45.7)	54.4% (54.0–54.8)	67.8% (67.3–68.3)
**Race/ethnicity**				
White	63.2% (62.2–64.3)	64.2% (63.2–65.3)	64.1% (63.0–65.2)	60.4% (59.1–61.7)
Black	19.6% (18.8–20.3)	20.7% (19.8–21.6)	20.3% (19.4–21.2)	22.0% (21.0–23.1)
Hispanic	11.3% (10.6–11.9)	9.5% (8.9–10.1)	9.5% (8.9–10.1)	10.3% (9.6–11.1)
Asian/Pacific islander	2.9% (2.6–3.2)	2.8% (2.5–3.1)	3.1% (2.8–3.6)	3.8% (3.4–4.4)
Native American	0.7% (0.6–0.8)	0.6% (0.6–0.7)	0.6% (0.6–0.7)	0.6% (0.5–0.7)
Other	2.3% (2.2–2.5)	2.2% (2.0–2.4)	2.4% (2.2–2.6)	2.8% (2.5–3.1)
**Primary payer**				
Medicare	71.0% (70.5–71.6)	75.8% (75.2–76.3)	76.4% (75.8–77.0)	73.9% (73.2–74.6)
Medicaid	10.3% (9.9–10.6)	9.0% (8.7–9.3)	8.7% (8.4–9.1)	9.9% (9.5–10.3)
Private insurance	14.2% (13.8–14.7)	11.1% (10.7–11.6)	10.8% (10.3–11.3)	12.0% (11.4–12.6)
Self-pay	1.8% (1.7–2.0)	1.5% (1.4–1.7)	1.5% (1.3–1.6)	1.6% (1.4–1.7)
No charge	0.1% (0.1–0.2)	0.1% (0.1–0.1)	0.1% (0.1–0.1)	0.1% (0.1–0.1)
Other	2.5% (2.3–2.7)	2.4% (2.3–2.6)	2.6% (2.4–2.8)	2.6% (2.4–2.8)
**Median household income (ZIP quartile)**				
Q1 (bottom 25%)	30.4% (29.4–31.4)	31.1% (30.1–32.2)	31.3% (30.2–32.3)	32.5% (31.3–33.7)
Q2	26.0% (25.3–26.7)	26.1% (25.4–26.9)	26.4% (25.6–27.1)	25.9% (25.1–26.7)
Q3	23.9% (23.3–24.6)	23.9% (23.2–24.7)	23.8% (23.1–24.6)	23.2% (22.4–24.0)
Q4 (top 25%)	19.6% (18.7–20.6)	18.8% (17.9–19.8)	18.5% (17.5–19.6)	18.4% (17.3–19.4)
**Hospital region**				
Northeast	18.1% (17.3–19.0)	17.6% (16.7–18.5)	17.0% (16.1–17.9)	16.6% (15.5–17.7)
Midwest	22.7% (21.8–23.7)	24.5% (23.5–25.6)	24.3% (23.2–25.5)	24.0% (22.7–25.5)
South	40.5% (39.5–41.6)	39.8% (38.7–40.9)	39.6% (38.4–40.8)	39.6% (38.1–41.0)
West	18.6% (17.8–19.5)	18.0% (17.2–18.9)	19.1% (18.2–20.0)	19.8% (18.7–21.0)
**Hospital teaching status**				
Rural	8.4% (7.9–8.9)	8.8% (8.3–9.4)	7.8% (7.2–8.4)	5.8% (5.2–6.4)
Urban non-teaching	16.6% (15.9–17.2)	16.8% (16.1–17.6)	16.3% (15.5–17.1)	15.0% (14.2–15.9)
Urban teaching	75.1% (74.3–75.9)	74.3% (73.5–75.2)	75.9% (75.0–76.8)	79.2% (78.2–80.2)
**Hospital bed size**				
Small	23.5% (22.7–24.4)	23.2% (22.3–24.1)	21.7% (20.7–22.7)	19.1% (18.0–20.2)
Medium	28.2% (27.3–29.1)	28.8% (27.9–29.8)	29.3% (28.3–30.3)	28.5% (27.3–29.7)
Large	48.3% (47.2–49.4)	48.0% (46.9–49.1)	49.0% (47.8–50.2)	52.4% (50.9–53.8)

**Table 3:** Adjusted associations between hemodynamic and vascular stressors and in-hospital outcomes.

Category	Mortality, aOR (95% CI)	AKI, aOR (95% CI)	LOS Ratio (95% CI)	Charge Ratio (95% CI)
**Stressor burden (ref = no stressors)**				
1	2.15 (2.08–2.23)	1.19 (1.18–1.20)	1.15 (1.14–1.15)	1.20 (1.19–1.21)
2	7.36 (7.09–7.64)	1.69 (1.66–1.71)	1.43 (1.42–1.44)	1.73 (1.72–1.75)
3+	31.6 (30.4–32.9)	2.99 (2.92–3.06)	2.08 (2.06–2.11)	3.36 (3.30–3.42)
**Stressor domain (ref = no stressors)**				
Hemodynamic only	4.97 (4.80–5.16)	1.48 (1.47–1.50)	1.25 (1.24–1.25)	1.39 (1.38–1.40)
Vascular only	2.15 (1.99–2.32)	0.92 (0.90–0.95)	1.17 (1.16–1.19)	1.33 (1.32–1.35)
Both	13.1 (12.5–13.7)	1.79 (1.75–1.83)	1.83 (1.81–1.85)	2.38 (2.34–2.42)

## Data Availability

The data that support the findings of this study are available from the Agency for Healthcare Research and Quality, Department of Health and Human Services of the United States. However, restrictions apply to the availability of these data, which were used under license for the current study, and so are not publicly available. Data are, however, available from the author upon reasonable request and with permission of the Agency for Healthcare Research and Quality.

## Supplementary Materials

The following supporting information can be downloaded at https://www.mdpi.com/article/10.3390/jcm15124747/s1. Table S1: ICD-10-CM Diagnosis Codes and ICD-10-PCS Procedure Codes Used in the Study; Table S2: Dose-Response Relationship Between Stressor Burden and Adverse Outcomes; Table S3: Sensitivity analysis excluding inter-hospital transfers; Table S4: Interaction between stressor burden and age group on in-hospital mortality.
