# Supplementary material for: Hemodynamic and Vascular Stressor Exposure and Outcomes Among Inpatient Hospitalization with Chronic Kidney Disease: A Nationwide Study
Source: J Clin Med. 2026 Jun 18;15(12):4747. doi: 10.3390/jcm15124747 (PMC13302244; doi:10.3390/jcm15124747)
Supplement: Supplementary file 1 [file jcm-15-04747-s001.zip › Supplementary Table S3.pdf]

Supplemental Table S3. Sensitivity analysis excluding inter-hospital transfers

Supplemental Table S3A. Stressor burden (Reference = 0 Stressors)

**Stressor category**	**Mortality, aOR (95% CI)**	**AKI, aOR (95% CI)**	**LOS ratio (95% CI)**	**Charge ratio (95% CI)**
1	2.17 (2.08–2.26)	1.16 (1.14–1.17)	1.15 (1.15–1.16)	1.20 (1.19–1.20)
2	7.77 (7.44–8.11)	1.62 (1.60–1.65)	1.46 (1.45–1.47)	1.73 (1.71–1.75)
≥3	34.99 (33.45–36.61)	2.87 (2.80–2.95)	2.14 (2.11–2.17)	3.33 (3.27–3.38)

Supplemental Table S3B. Stressor domain (Reference = No Stressors)

**Stressor domain**	**Mortality, aOR (95% CI)**	**AKI, aOR (95% CI)**	**LOS ratio (95% CI)**	**Charge ratio (95% CI)**
Hemodynamic only	5.13 (4.92–5.34)	1.36 (1.35–1.38)	1.25 (1.25–1.26)	1.38 (1.37–1.39)
Vascular only	2.21 (2.02–2.41)	0.83 (0.80–0.85)	1.17 (1.16–1.18)	1.34 (1.32–1.35)
Both	14.13 (13.45–14.85)	1.62 (1.58–1.66)	1.85 (1.82–1.87)	2.35 (2.32–2.39)

Supplemental Table S3 shows sensitivity analysis excluding inter-hospital transfers examining the association between hemodynamic and vascular stressor burden and in-hospital outcomes among hospitalized adults with chronic kidney disease. Adjusted odds ratios (aORs) and ratios with 95% confidence intervals are shown for stressor burden and stressor domain categories. Models excluded inter-hospital transfers and were adjusted for age, sex, race/ethnicity, primary payer, ZIP-code income quartile, hospital region, teaching status, bed size, ownership, and elective admission status.
