# Supplementary material for: Hemodynamic and Vascular Stressor Exposure and Outcomes Among Inpatient Hospitalization with Chronic Kidney Disease: A Nationwide Study
Source: J Clin Med. 2026 Jun 18;15(12):4747. doi: 10.3390/jcm15124747 (PMC13302244; doi:10.3390/jcm15124747)
Supplement: Supplementary file 1 [file jcm-15-04747-s001.zip › Supplementary Table S4.pdf]

Supplemental Table S4. Interaction between stressor burden and age group on in-hospital mortality

**Stressor Burden**	**Age <65, aOR (95% CI)**	**Age ≥65, aOR (95% CI)**	**p for interaction**
1	2.96 (2.69–3.26)	2.05 (1.97–2.13)	
2	14.59 (13.26–16.06)	6.43 (6.18–6.70)	P<0.001
≥3	67.68 (61.53–74.44)	25.59 (24.51–26.72)	P<0.001

Supplemental Table S4 shows the interaction between hemodynamic and vascular stressor burden and age group on in-hospital mortality among hospitalized adults with chronic kidney disease. Adjusted odds ratios (aORs) and 95% confidence intervals are shown separately for patients aged <65 years and ≥65 years. Interaction p-values were derived from survey-weighted logistic regression models including a stressor burden × age group interaction term. Models were adjusted for sex, race/ethnicity, primary payer, ZIP-code income quartile, hospital region, teaching status, hospital bed size, ownership, elective admission status, and transfer status.
