# Supplementary material for: Hemodynamic and Vascular Stressor Exposure and Outcomes Among Inpatient Hospitalization with Chronic Kidney Disease: A Nationwide Study
Source: J Clin Med. 2026 Jun 18;15(12):4747. doi: 10.3390/jcm15124747 (PMC13302244; doi:10.3390/jcm15124747)
Supplement: Supplementary file 1 [file jcm-15-04747-s001.zip › Supplementary Table S1.pdf]

Supplemental Table S1. ICD-10-CM Diagnosis Codes and ICD-10-PCS Procedure Codes Used in the Study

**Variable / Construct**	**Category**	**ICD-10-CM / ICD-10-PCS Codes**	**Notes**
Chronic kidney disease (CKD)	Study population	N18.*	Identified in any diagnosis position
Sepsis	Hemodynamic stressor	A40.*, A41.*, R65.20, R65.21	Includes sepsis and septic shock
Shock	Hemodynamic stressor	R57.*	Includes cardiogenic, septic, hypovolemic, and other shock
Acute myocardial infarction (AMI)	Vascular stressor	I21.*, I22.*	Acute ST-elevation and non-ST-elevation MI
Acute decompensated heart failure (ADHF)	Hemodynamic stressor	I50.*	Includes acute and acute-on-chronic heart failure
Major bleeding	Vascular stressor	K92.2, D62, I85.01, I85.11, K25.0–K28.2	Includes gastrointestinal and major hemorrhage with blood loss anemia
Mechanical ventilation	Hemodynamic stressor	5A1935Z, 5A1945Z, 5A1955Z	ICD-10-PCS procedure codes
Stroke	Vascular stressor	I60.*, I61.*, I63.*, I64	Includes ischemic and hemorrhagic stroke
Pulmonary embolism (PE)	Vascular stressor	I26.*	Acute pulmonary embolism
Deep vein thrombosis (DVT)	Vascular stressor	I82.*	Includes proximal and distal DVT
Acute kidney injury (AKI)	Outcome	N17.*	Identified in any diagnosis position
In-hospital mortality	Outcome	NIS variable: DIED = 1	Administrative discharge disposition

Variable / Construct	Category	ICD-10-CM / ICD-10-PCS Codes	Notes
Inter-hospital transfer	Sensitivity analysis	NIS variables: TRAN_IN = 1 or TRAN_OUT = 1	Used to exclude transfers in sensitivity analyses

Supplemental Table S1 shows the ICD-10-CM diagnosis codes and ICD-10-PCS procedure codes used to define chronic kidney disease, acute hemodynamic and vascular stressors, outcomes, and sensitivity analyses. Codes were identified in any diagnosis or procedure position unless otherwise specified.
