# Supplementary material for: Hemodynamic and Vascular Stressor Exposure and Outcomes Among Inpatient Hospitalization with Chronic Kidney Disease: A Nationwide Study
Source: J Clin Med. 2026 Jun 18;15(12):4747. doi: 10.3390/jcm15124747 (PMC13302244; doi:10.3390/jcm15124747)
Supplement: Supplementary file 1 [file jcm-15-04747-s001.zip › Supplementary Table S2.pdf]

Supplemental Table S2. Dose-Response Relationship Between Stressor Burden and Adverse Outcomes

**Outcome**	**Adjusted OR/Ratio per 1-Category Increase in Stressor Burden (95% CI)**	**P for Trend**
In-hospital mortality	3.42 (3.38–3.47)	<0.001
Acute kidney injury	1.35 (1.34–1.36)	<0.001
Length of stay	1.24 (1.23–1.24)	<0.001
Hospital charges	1.40 (1.40–1.41)	<0.001

Supplemental Table S2 shows dose-response analyses evaluating stressor burden as an ordinal variable (0, 1, 2, and ≥3 stressors). Estimates represent the adjusted odds ratio (mortality and AKI) or adjusted ratio (length of stay and hospital charges) associated with each one-category increase in stressor burden. Models were adjusted for age, sex, race/ethnicity, primary payer, ZIP-code income quartile, hospital region, teaching status, hospital bed size, hospital ownership, elective admission status, and transfer status.
